# Evaluating personnel evacuation risks under fire scenario of Airbus wide-body aircraft: A simulation study

**DOI:** 10.3389/fpubh.2022.994031

**Published:** 2022-09-08

**Authors:** Wei Lv, Luliang Xing, Jiawei Li, Caihong Zhao, Yunpeng Yang

**Affiliations:** ^1^School of Safety Science and Emergency Management, Wuhan University of Technology, Wuhan, China; ^2^Saifeite Engineering Technology Group Co., Ltd., Qingdao, China

**Keywords:** Airbus aircraft, airliner environment, personal safety, evacuation risk, fire exposure

## Abstract

Airliner accidents are often accompanied by incidental aircraft fires, causing huge casualties and incalculable economic losses. The research on airliner fire and its emergency evacuation is the focus and difficulty of aviation safety research, but it is difficult to carry out the research through experiments, and the use of computer simulation is an effective method. This paper comprehensively studies the dynamic development of the cabin fire and the corresponding cabin evacuation when the wide-body airliner Airbus A350-900 is forced to land in two states: horizontal and forward. The spatial distribution of the remaining evacuation time at each seat is used to analyze and judge the safety evacuation risk of the airliner cabin. Finally, two evacuation optimization design ideas based on partition guidance and seat layout are proposed to improve the spatial distribution of the overall evacuation risk of passengers in the cabin and provide some reference suggestions for strengthening fire prevention in the design, manufacture, and use of airliner. Some targeted countermeasures are put forward for the emergency evacuation of passengers in the cabin in a fire situation.

## Highlights

- The influence of the capacity of aircraft emergency slides on passenger evacuation is considered.
- A spatial representation method of personnel evacuation risk is proposed.
- An unconventional working condition is analyzed when the aircraft is tilted forward.
- An optimization idea is proposed to optimize the distribution of remaining evacuation time.

## Introduction

A survey of air accidents in the past 30 years found that in civil aviation, fires often occur near the landing gear, cabins, and hangars and are often extremely sudden. The causes of fire accidents generally include fuel leakage, landing impact fire of passenger aircraft, the fire of dangerous goods in luggage, short circuit fire of electrical system, and an engine fire caused by improper maintenance of passenger aircraft (1). If a fire occurs inside the cabin, such an accident is extremely unfavorable for the evacuation of passengers. For the research on cabin fire, most scholars often use empirical formulas to calculate the fire development process, use CFD technology to analyze the temperature field distribution, or use advanced fire extinguishing equipment to evaluate the fire extinguishing effect; at the same time, a few scholars use full-size or small-size aircraft to carry out In experimental research, some scholars use professional software numerical simulation tools to study fire. Most of these studies are based on simple physical models.

As early as 1976, the National Aeronautics and Space Administration carried out the physical fire resistance experiment on passenger aircraft (2). In 1979, the Federal Aviation Administration of the United States put aviation fuel outside the passenger cabin door to conduct combustion experiments to analyze the impact of fire on various parameters in the passenger cabin (3). The National Aeronautical Facility Test Center in the United States conducted a related aircraft fire experiment in 1996. Researchers used a full-size transport aircraft to transform its cabin into an airliner cabin (4).

Wang (1) selected the Airbus A330-300 civil passenger aircraft as the research object in 2016 and used the modeling method to simulate and analyze the dynamic development law of the fire of the Airbus A330-300 passenger aircraft. In 2019, Bai (5) conducted a risk assessment on a large passenger aircraft, the Airbus A330-300 passenger aircraft, and proposed a specific fire risk assessment method for passenger aircraft. Zhang et al. (6) used the fire simulation software PyroSim in 2012 to simulate the fire scene in an airtight passenger cabin and obtain the simulation results for further analysis.

Liu (7) researched and collected information such as the cabin's plane layout, established a mathematical model that restores the real situation as much as possible based on this information, and finally obtained the model with the fastest evacuation speed. Yu (8) and others established a passenger aircraft cabin evaluation system with 5 indicators and 23 influencing factors as the main content and blushed effective, comprehensive evaluation methods for evacuation capabilities. Fu (9) used Pathfinder software to simulate the evacuation of Boeing 777-200 aircraft by setting 5 specific scenarios.

From the above summary, it can be seen that domestic and foreign scholars have carried out extensive research on aircraft fires and personnel emergency evacuation, and the research has continued to deepen. The research mainly focuses on mathematical reasoning, simulation, and experimental analysis of aircraft fire and passenger evacuation.

### Modeling

**Figure 1:**
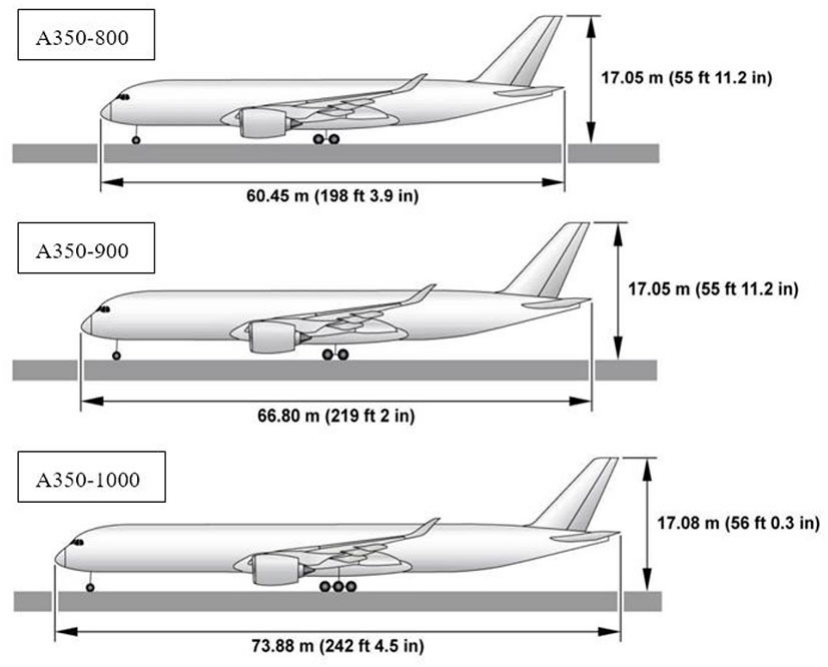
Airbus A350 series model appearance and size comparison.

**Figure 2:**
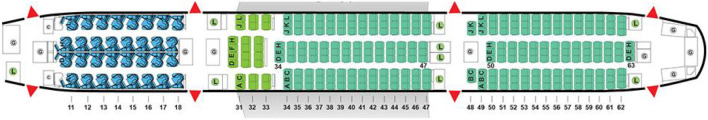
Plane layout of Airbus A350-900 passenger cabin.

Airbus manufactures the A300, A310, A318, A319, A320, A321, A330, A340, A350, A380, and other aircraft. Among them, the A350 series consists of A350-800, A350-900, and A350-1000, which are small to large and carry 270, 312, and 350 passengers, respectively, as shown in Figure 1. In this paper, the Airbus A350-900, a large twin-engine ultra-long-range, two-aisle wide-body airliner, is selected as the object of study. The cabin layout of this type of airliner is shown in Figure 2.

### Basic parameters of the model

After collecting and sorting out, the main basic parameters of Airbus A350-900, such as fuselage length, fuselage height, cabin length, and cabin width, are shown in Table 1.

**Table 1:** Main parameters of Airbus A350-900.

**Parameter**	**Value**	**Parameter**	**Value**
Length (m)	66.80	Length of cabin (m)	51.04
Height of fuselage (m)	6.09	Width of fuselage (m)	5.96
Height of cabin (m)	2.2	Width of cabin (m)	5.61
Height of airliner (m)	17.05	Number of doors	8
Wingspan (m)	64.75	Wheelbase (of a vehicle) (m)	28.66

In the passenger cabin of Airbus A350-900, the seating arrangement is a typical three-class cabin arrangement consisting of business class, super economy class, and economy class. Its seating arrangement is as follows: rows 11–18 are business class, with a total of 32 seats; rows 31–33 are super economy class, with a total of 24 seats; rows 34–63 are economy class, with a total of 256 seats. The basic parameters of specific cabin seats are shown in Table 2 below.

**Table 2:** Basic parameters of cabin interior seats.

**Class of cabin**	**Business class**	**Premium economy class**	**Economy class**
Number of seats	32	24	256
Row of seats	8	3	30
Seat pitch (m)	1.09	0.97	0.81
Width of seat (m)	0.58	0.48	0.44
Maximum seat reclining angle (°)	180	120	100

### Design of fire scene

When building a fire scenario, the known conditions and potential risks should be taken into account, the parameters of the airliner should be understood, the safety influencing factors in the cabin should be identified, and the location of the fire source should be reasonably set around the purpose of the study (10). Civilian airliners generally have a central fuel tank or additional central fuel tank near the center of gravity of the fuselage. As early as 1989, the Civil Aviation Administration of the United States carried out a physical simulation experiment of cabin fire (1). The simulation scenario is: after the plane fell, the huge impact force destroyed the structure of the plane, the fuel tank on one side of the wing ruptured, and the fuel leaked, causing the external fuel to form a pool fire and then cause a cabin fire through thermal radiation.

This paper investigates the fire caused by the impact of a civil airliner on the ground causing damage to the front section structure of the main body of the airliner, resulting in the spillage of aviation fuel carried in the additional central tank of the airliner due to the cracking of the cabin. Further, it investigates the impact of the fire on the evacuation of passengers in the passenger cabin of the airliner. The fire scenario is designed around the issue of cabin passenger evacuation, and the fire scenario is designed by considering the maximum probability principle and the least favorable principle through literature research on airliner fires.

The air pressure in the cabin was set to 1 standard atmosphere, and the initial temperature was set to 20°C. The Airbus A350-900 wide-body airliner studied in this paper is a large airliner with a long fuselage and a large cabin space. The passenger cabin is a relatively closed, narrow, and long cavity. The external environment affects only a small area near the door and does not significantly affect the fire development in the entire cabin. In the standard case, the property parameters of aviation fuel are in Table 3.

**Table 3:** Main property parameters of aviation fuel (11, 12).

**Parameter**	**Heat of combustion (MJ·kg^−1^)**	**Progressive combustion efficiency (kg·m^−2^·s^−1^)**	**Chemical combustion efficiency**	**Density (kg·m^−3^)**	**Effective absorption coefficient (L·m^−1^)**
Aviation fuel	43	0.035	0.9	940	1.7

The cabin bulkhead structure of civil airliner is often composed of aluminum alloy, which is flame retardant. To simplify this paper, the passenger cabin bulkhead is set to aluminum, and other materials such as seats are also made flame-retardant, and in the standard case, the property parameters of aluminum are shown in Table 4.

**Table 4:** Main property parameters of aluminum (13).

**Parameter**	**Value**	**Parameter**	**Value**
Density (kg/m^3^)	2700	Thermal conductivity [W/(m·k)]	237
Specific heat capacity [J/(kg·°C)]	880	The heat of evaporation (J/kg)	1.05 × 10^7^
Melting point (°C)	660.4	Boiling point (°C)	2467
The heat of dissolution (J/kg)	3.98 × 10^5^	Emissivity	0.9

**Figure 3:**
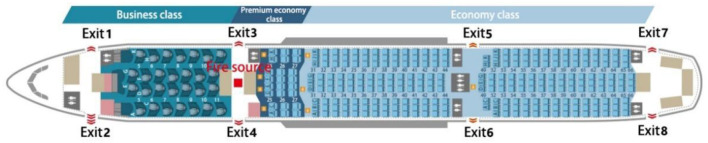
Diagram of each Exit number and the location of the fire source in the passenger compartment.

The size of the fire source is set to a rectangular oil pool of 2.0 × 2.0 m. The fire location is in the middle of the second pair of evacuation exits connected to the passenger cabin near the front side of the nose, with the red square representing the fire source. In contrast, the 4 pairs of doors of the airliner are numbered for the convenience of description, with the doors near $Q_{\max}=m^{\prime \prime }\Delta H_{c}\chi \pi D^{2}/4$ the nose side numbered Exit 1 and Exit 2, the doors in the middle section numbered Exit 3 to Exit 6, and the doors near the tail side numbered Exit 7 and Exit 8, as shown in Figure 3.

**Figure 4:**
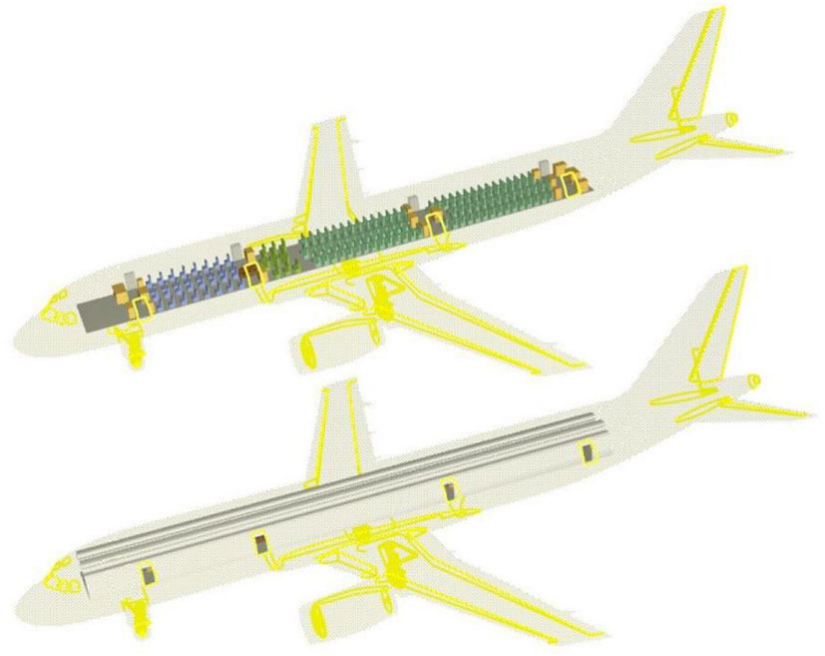
3D view of the PyroSim model of the Airbus A350-900 aircraft and cabin.

According to the plane layout of the Airbus A350-900 airliner, the three-dimensional model of the cabin can be established after reasonably simplifying the model, as shown in Figure 4.

### Fire source parameter settings

In aviation accidents, once the oil tank is damaged and leaked, it is easy to cause oil injection or dripping, and an oil pool will be formed. The fire caused by aircraft fuel leakage can be divided into oil vapor fire, jet fire, and oil pool fire according to the fuel combustion mode. In practice, the combustion mode of fuel oil may be single or composite, which constitutes the complexity of cabin fire. In this paper, to simplify the fire calculation, only pool fire is considered for calculation. The calculation formula for the maximum heat release rate of oil pool fire is as follows (11):


(1) $$\begin{array}{r} Q_{max}=m^{\prime \prime }\Delta H_{c}\chi^{\pi }D^{2}/4 \end{array}$$

Where, Δ*H*_*c*_ is the combustion heat, kJ·kg^−1^; χ is the combustion efficiency; *D* is the diameter of the oil pool, m; *m*″is the mass combustion efficiency per unit surface area, kg·m^−2^·s^−1^; The calculation formula is:


(2) $$\begin{array}{r} m^{\prime \prime }={m_{\infty }}^{\prime \prime }\left(1-e^{K\prime D}\right) \end{array}$$

In the formula, ${m_{\infty }}^{\prime \prime }$ is the progressive mass burning rate of large-scale oil pool fire, kg·m^−2^·s^−1^; *k*′ is the effective absorption coefficient.

The fire source in the model is set in the middle of the connecting line between the second pair of hatch doors (Exit 3 and Exit 4) on the front side of the airliner. The rectangle with the size of 2.0 × 2.0 m is regarded as pool fire combustion. The fuel volume is assumed to be 0.1 m^3^, and the following formula can calculate its equivalent diameter:


(3) $$\begin{array}{r} D=\sqrt{4S/pi} \end{array}$$

Where *S* is the area of the liquid pool, according to the above, it can be calculated that the maximum heat release rate of the oil pool fire in this paper is 5.3011 MW.

**Figure 5:**
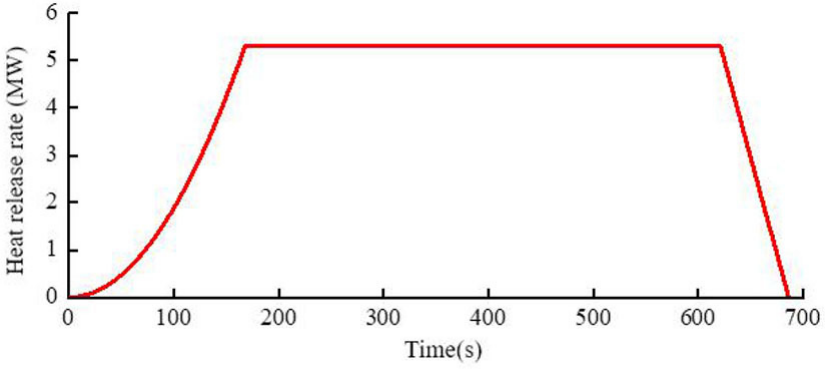
Growth curve of heat release rate.

The model in this paper sets the fire type as a very fast-growing fire, and the corresponding fire growth coefficient α = 0.1878 kW/s^2^. According to the formula, it can be calculated that the time when the maximum heat release rate is reached is 168 s, and the combustion efficiency per unit surface area of the fuel is calculated to be 0.03424 kg·m^−2^·s^−2^, from the total volume of fuel in the pool fire and the mass combustion efficiency of the fuel, it can be calculated that the burning time of the pool fire is 686 s. The decline of sump burning is a relatively complex process. The time when the fire source begins to decline is affected by the fire structure and other factors, and it is difficult to obtain an accurate solution. The decay rate of most fuels is 0.0001 m/s, and at some point the fuel is consumed to the point where the combustion cannot support the maximum heat release rate and begins to decay. At this time, the heat radiation is generally higher, and the fire decay process is faster. The schematic diagram of the fire scene's heat release rate development curve in this paper is shown in Figure 5.

FDS software simulates and calculates the CO concentration and flue gas diffusion according to the chemical equivalent combustion reaction of the specified fuel. Setting the co-production rate, flue gas production rate, and other parameters is necessary. Aviation fuel is a complex mixture, and aviation kerosene was chosen as a typical fuel for simplicity. Under standard conditions, its thermophysical parameters are as follows (14). The contents in brackets are the interpretation of the command line:

&REAC ID = ‘KEROSENE' (The fuel is called fuel oil)

FYI = ‘Kerosene, C_14 H_30, SFPE Handbook' (The molecular formula is C_14_H_30_)

MW_FUEL = 198.0 (The molar mass is 198)

NU_O2 = 21.5 (The coefficient of O_2_ is 21.5)

NU_CO2 = 14.0 (The coefficient of CO_2_ is 14)

NU_H2O = 15.0 (The coefficient of H_2_O is 15)

EPUMO2 = 12700 (The energy released per unit mass of oxygen is 12700 kJ/kg)

CO_YIELD = 0.012 (The CO generation rate from combustion is 0.012)

SOOT_YIELD = 0.042/(The soot generation rate is 0.042)

**Figure 6:**
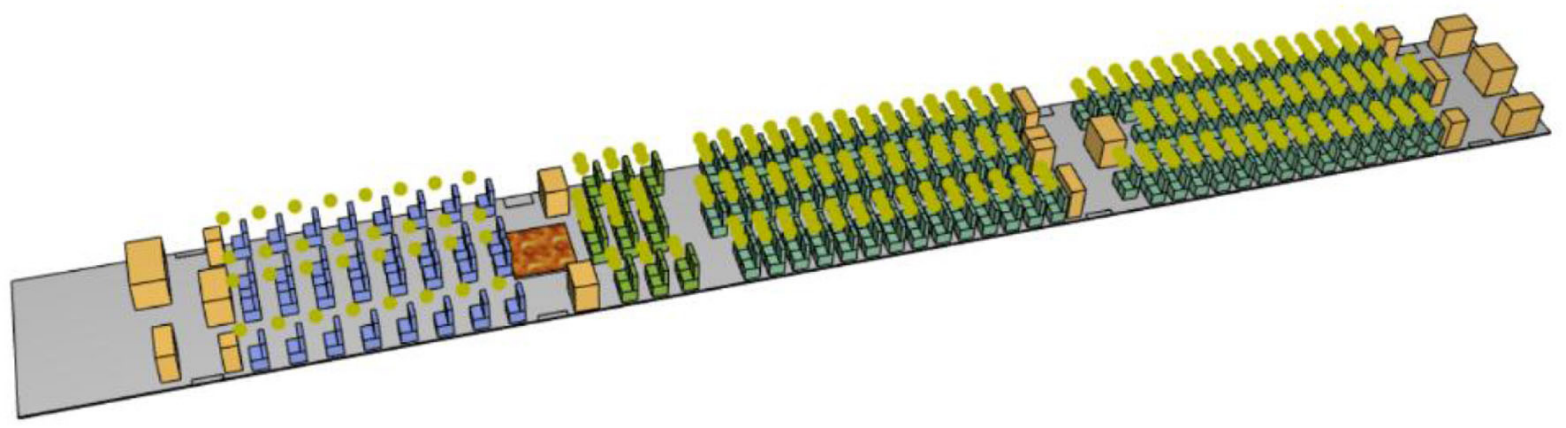
Location diagram of 312 observation points of the cabin model.

The survey found that most passengers of medium and long-range wide-body airliners are adults. The average height of adults in China is about 1.70 m. It can be considered that the mouth and nose of passengers in the cabin during evacuation movement are about 1.65 m from the cabin ground. Therefore, this paper takes the cabin ground as the reference plane (H = 0 m), and the monitor is set at the height of H = 1.65 m, which is arranged concerning the layout of various types of seats in the cabin. A total of 312 observation points are set in the model. The positions of each observation point are shown in Figure 6. In the figure, the left side is the nose side of the aircraft, the observation points in business class are numbered from observation point 1 to observation point 32, and the observation points in super economy class are numbered from observation point 33 to observation point 56, and the observation points in economy class are numbered from observation point 57 to observation point 312. The numerical CO concentration, temperature, and visibility changes can be measured at each observation point. In the following chapters of this paper, these 312 observation points are selected to evaluate the safety indexes near all types of seats of the Airbus A350-900 wide-body airliner.

When a fire occurs, there will be many adverse factors that will cause harm to the human body. In this paper, the commonly used indicators in the fire are selected to analyze the available safe evacuation time of cabin fire. To ensure that passengers in the cabin can complete safe evacuation in the case of a cabin fire, the following specific quantitative standards are set as the critical value of danger for further analysis:

(1) The CO concentration in the passenger cabin at the height of 1.65 m from the ground is <50 ppm, which is converted into 0.00005 mol/mol commonly used in Pyrosim software;
(2) The flue gas temperature in the passenger cabin at the height of 1.65 m from the ground is <60°C;
(3) The visibility of the passenger cabin at the height of 1.65 m from the ground is >5 m.

## Simulation and results

### Fire simulation and analysis

After investigation, it is found that the forced landing of passenger aircraft is often accompanied by incidental fire accidents, and the passenger aircraft is often in a horizontal and inclined state during the forced landing. Among them, the front landing gear of the passenger aircraft bears a huge impact force when landing, and the phenomenon of failure is not uncommon. When this happens, the passenger plane is often prone to lean forward, and the dynamic development of the fire at this time is also quite different from that when the passenger plane is in a horizontal and normal state. Therefore, it is necessary to carry out a comparative study of the two states.

**Figure 7:**
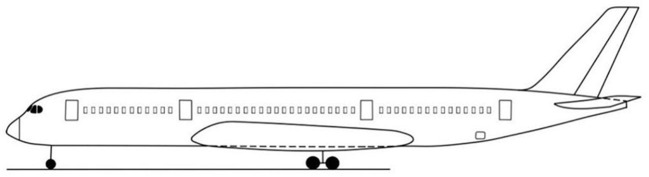
Schematic diagram of an airliner in the horizontal state.

When the airframe of the airliner is functioning normally, the front and rear landing gears are not damaged and can work normally, and the pilot drives the airliner properly so that the airliner is forced to land normally without tilting. As shown in Figure 7, the airliner remains horizontal after the forced landing. The length of the simulation time considers the specific simulation objects and scenarios. According to the relevant airworthiness regulations and practical experience of civil aviation aircraft, and this paper studies the passenger evacuation problem in the cabin fire scenario, the fire simulation time can be set as 200 s.

To obtain the ASET at each seat in the cabin, it is also necessary to calculate and analyze the time for the three indicators of CO concentration, temperature, and visibility to reach the dangerous critical value at the height of 1.65 m at the 312 observation points above the 312 seats under the two states of the airliner (15, 16). To select the time when any of the three safety evaluation indicators at each observation point first reaches the critical value of danger as the available safe evacuation time for the observation point.

**Figure 8:**
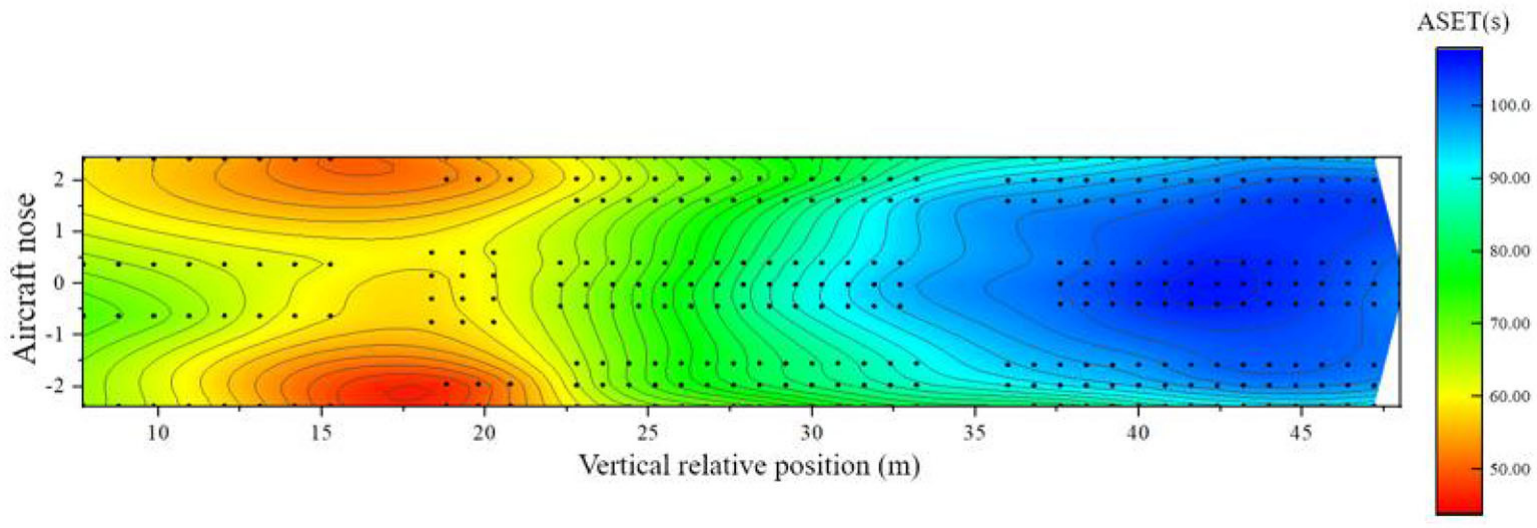
Distribution of available evacuation time under horizontal cabin state.

When the airliner is in a horizontal state, the minimum time when any of the three safety indicators at each seat of the airliner reaches the critical value is calculated as the available safe evacuation time there, and the spatial distribution map of the airliner in the horizontal state is drawn, as shown in Figure 8.

From Figure 8, the distribution of ASET in the horizontal state of the passenger aircraft is analyzed as follows:

(1) Within the area of 6–12 m relative to the longitudinal position of the cabin: this area is mainly business class. Since this area is relatively close to the fire source, it is significantly affected by the fire, and the safe evacuation time is within 70 s. The seat temperature near the sidewall of the cabin reaches the critical value first, and the ASET is shorter;
(2) Within the 12–23 m area relative to the longitudinal position of the cabin: this area contains a small number of business class seats and all super economy class seats. The fire source is in this area, which is most affected by the fire. The available safe evacuation time of each seat is almost all within 60 s, and the temperature and visibility of most areas reach the critical value first;
(3) Within the area of 23–34 m relative to the longitudinal position of the cabin: this area includes all the economy class seats set in the middle of the cabin. The area affected by the fire gradually decreases from the front side to the rear side, the available safe evacuation time ranges from 60 to 90 s, and most areas are also the first to reach the critical value of temperature and visibility;
(4) Within the area 34 m behind the longitudinal relative position of the cabin: this area includes all the economy class seats arranged in the rear section of the cabin. Since this area is the farthest from the fire source, it is relatively less affected by the fire. Except for a few seats near the sidewall of the cabin, the available evacuation time is shorter, and the ASET of the rest of the seats is >90 s. The seats in this area are generally safe. The visibility index is the first to reach the critical value of danger.

**Figure 9:**
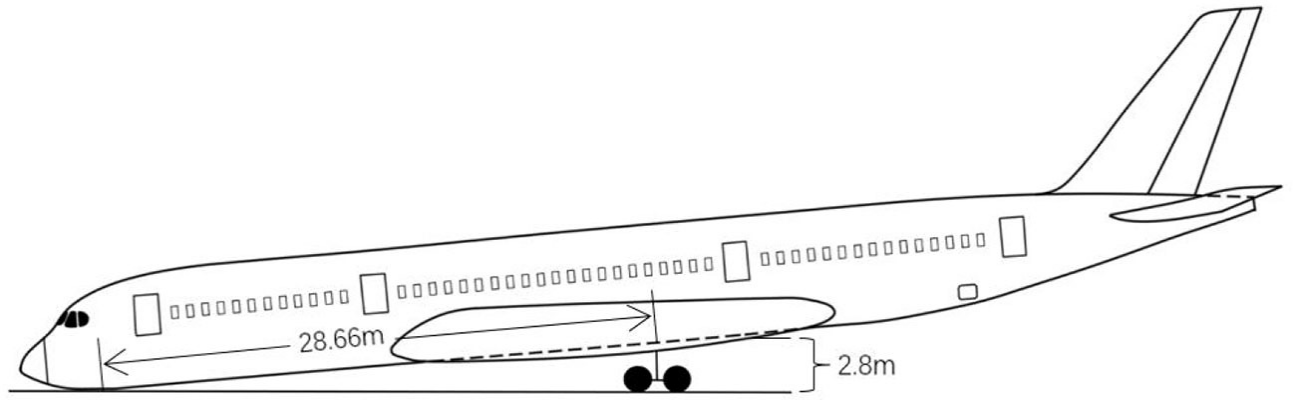
Schematic diagram of the passenger aircraft in a forward-tilted state.

When the airliner is forced to land, if the pilot does not operate properly and the uncertainty of the forced landing position is added, the probability of collision damage and failure of the nose landing gear is very high. The Airbus A350-900 aircraft studied in this paper adopts the front three-point landing gear design. The disadvantage of this landing gear design is that the front landing gear bears a large load. If the aircraft adopts an abnormal landing attitude, it can easily cause damage and failure. The passenger will lean forward, as shown in Figure 9.

**Figure 10:**
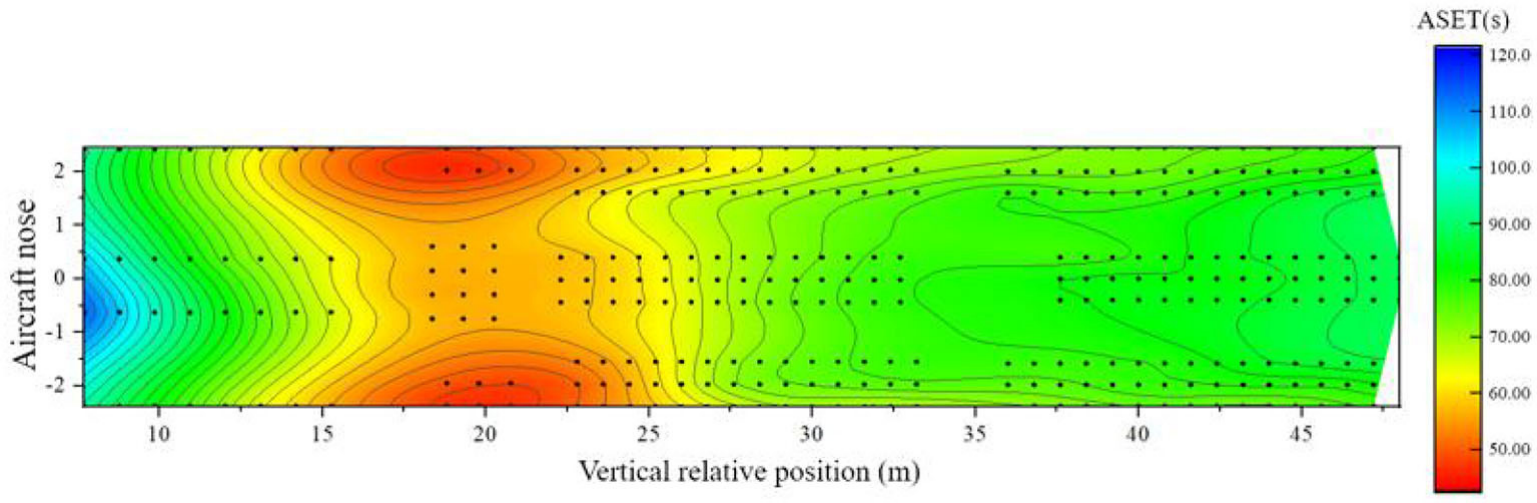
Distribution of available evacuation time when the cabin is tilted forward.

Similarly, the distribution of ASETs around the cabin when the aircraft is tilted forward can be calculated, as shown in Figure 10.

From Figure 10, the analysis of the ASET distribution in the forward tilt state of the passenger aircraft is as follows:

(1) Within the 6–13 m area relative to the longitudinal position of the cabin: this area is mainly for most of the seats in business class. Although this area is relatively close to the fire source, under the influence of the forward tilt of the airliner, the available safe evacuation time is almost always >70 s, which is improved compared with the horizontal state. The available evacuation time for a small number of seats closest to the nose side is >100 s. The temperature of most seats in this part of the area is the first to reach the dangerous threshold;
(2) Within the 13–26 m area relative to the longitudinal position of the cabin: this area contains a small number of business class seats, all super economy class seats, and a small number of economy class seats set in the middle of the cabin. The fire source is located in this area, the most significant area in the cabin affected by the fire. Each seat's available safe evacuation time is mostly within 60 s, and the ASET of the seats near the bulkhead is <50 s. The indicator reaches the dangerous threshold very quickly;
(3) Within the 26–35 m area relative to the longitudinal position of the cabin: this area includes most of the economy class seats set in the middle of the cabin. The influence degree of the area affected by the fire from the front side to the backside is not obvious compared with the horizontal state, and the available safe evacuation time ranges from 60 to 80 s, and most areas are also the first to reach the critical value of temperature and visibility;
(4) Within the area 35 m behind the longitudinal relative position of the cabin: this area includes all the economy class seats set in the rear section of the cabin. Since this area is the farthest from the fire source, the available evacuation time is relatively long, almost all-around 80 s. Due to the influence of the forward tilt, it is reduced compared with the horizontal state, and the temperature and visibility are the first to reach the critical value of danger.

### Evacuation simulation and analysis

1. Setting of evacuation scenarios
  Through the analysis of the previous fire development results, it can be seen that after the emergency landing of the airliner and the fire occurs, the CO concentration, temperature, and visibility in the cabin are quite different when the cabin is horizontal and forward. Walking speed is also affected. When simulating cabin evacuation in this paper, two scenarios are set accordingly:
  (1) Scenario 1: In the fire scenario when the cabin is in a horizontal state after a forced landing, the evacuation scenario in which all passengers are at their seats when the cabin is fully loaded;
  (2) Scenario 2: In the fire situation when the cabin is in a forward-tilted state after the forced landing, the evacuation scene is in which all passengers are in their seats when the cabin is fully loaded.
2. Setting of cabin door conditions
  In this paper, the forced landing of the passenger aircraft is considered to be in a horizontal forward-tilted state. Under this emergency evacuation condition, all cabin doors are considered to be able to open normally and immediately. In this model, the cabin is arranged with 8 cabin doors. However, corresponding to the maximum probability principle and the most unfavorable principle above, the fire occurred the Exit 3 and Exit 4 of the aircraft cabin near the nose side. It is assumed that people cannot be evacuated through Exit 3 and Exit 4, and the airliner is not level or forward. Cause other doors to fail, so this paper studies the situation where the other 6 Exits can be used for evacuation. For a large wide-body passenger aircraft with a huge passenger capacity, this situation is extremely unfavorable for evacuation. The default direction is the direction the seat is facing.
3. Setting of model personnel parameters
  When using the software to simulate the evacuation of passengers in the cabin, the personnel attributes should be highlighted as much as possible to ensure that the simulation results are closer to reality. Generally, different genders and ages are often used in Pathfinder software to define different categories of personnel. In this paper Personnel, shoulder width is set to 40 cm. After a comparative analysis of the quarterly operating data and passenger turnover and other information released by a civil aviation airline, it was found that the ratio of male to female passengers was close to 6:4, and the age range: was 30–50 years old accounted for about 70%; under 30 years old accounted for 20% about 10%; the rest account for about 10%. The maximum passenger capacity of the airliner is 312 people, according to which the personnel is divided as follows. See Table 5 for details.
  This paper believes that the main factors affecting the walking speed of people on the ground in the cabin are age and gender. After consulting, it was found that there is no official approved clear regulation on the walking speed of evacuees in China. Therefore, this paper refers to the relevant empirical formulas summarized by some foreign scholars for estimation (17–21). Scholars summarized the relationship between people's walking speed on flat ground and age and gender. Generally speaking, people aged 29 and below walk faster. When the age is the same, the walking speed of men is often higher than that of women. The specific empirical formula of walking speed on flat ground is shown in Table 6.
  Referring to the above empirical formula, the walking speeds of people of different ages and genders in the passenger cabin passageway can be approximately estimated, respectively, as shown in Table 7.
  In the evacuation situation when the airliner is tilted forward by 5.6°, due to the complicated cabin conditions, how to change the overall speed of the personnel has become a more complicated problem. In this article, we reduce the minimum individual speed by 10%, increase the maximum individual speed by 5%, and the average moving speed of passengers is appropriately reduced to simplify processing, as shown in Table 8.
4. Cabin exit settings considering the capacity of aircraft slides
  The passenger cabin of a passenger aircraft is a long and narrow cavity, the ground in the cabin is flat, and the evacuation path is relatively simple. It's the dual aisle in the cabin. The evacuation Exits are symmetrically distributed on the left and right sides of the cabin, with a total of 8, and the width of each Exit is 0.9 m.

**Table 5:** Age and gender settings of evacuees.

**Passenger**	**Number**	**Proportion (%)**
**category**	**of passengers**	
Women aged 29 and younger	25	8.01
Men aged 29 and younger	37	11.90
Women aged 30–50	87	27.90
Men aged 30–50	131	41.93
Women 51 and older	13	4.17
Men 51 and older	19	6.09
Total	312	100

**Table 6:** The empirical formula for the walking speed of personnel on the level ground (22).

**Gender**	**Age (*n*)**	**Walking speed (m/s)**
Women	2–8.3	0.06n + 0.5
	8.3–13.3	0.04n + 0.67
	13.3–22.25	0.02n + 0.94
	22.25–37.5	– 0.018n + 1.78
	37.5–70	– 0.01n + 1.45
Men	2.0–5.0	0.16n + 0.3
	5–12.5	0.06n + 0.8
	12.5–18.8	0.008n + 1.45
	18.8–39.2	– 0.01n + 1.78
	39.2–70	– 0.09n + 1.75

**Table 7:** The walking speed of different groups of people in the aisle when the cabin is horizontal (23).

**Passenger**	**Cabin aisle personnel**		
**category**	**walking speed**		
	**Minimum**	**Maximum**	**Average**
	**speed**	**speed**	**speed**
Women aged 29 and younger	0.91	1.63	1.27
Men aged 29 and younger	1.08	1.81	1.45
Women aged 30–50	0.78	1.28	1.03
Men aged 30–50	0.92	1.66	1.29
Women 51 and older	0.58	0.97	0.78
Men 51 and older	0.88	1.45	1.17

**Table 8:** The walking speed of different groups of people in the aisle when the cabin is tilted forward.

**Passenger**	**Cabin aisle personnel**		
**category**	**walking speed**		
	**Minimum**	**Maximum**	**Average**
	**speed**	**speed**	**speed**
Women aged 29 and younger	0.82	1.71	1.22
Men aged 29 and younger	0.97	1.90	1.39
Women aged 30–50	0.70	1.34	0.99
Men aged 30–50	0.83	1.74	1.25
Women 51 and older	0.52	1.02	0.74
Men 51 and older	0.79	1.52	1.12

**Figure 11:**
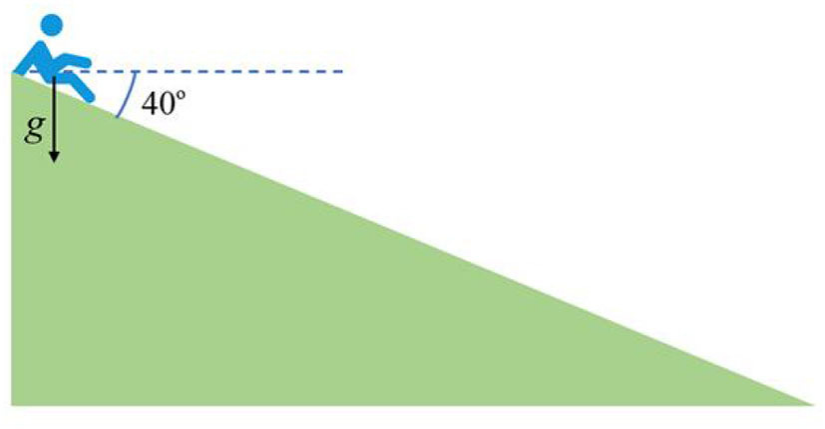
Equivalent schematic diagram of the escape slides at each Exit when the airliner is level.

In the emergency evacuation scenario of a real aircraft forced landing, passengers need to be evacuated from the Exit of the cabin door through the escape slide. Therefore, the evacuation efficiency of passengers is closely related to the capacity of the escape slide. The evacuation flow of the escape slide is generally smaller than that of the cabin door, which will cause passengers to wait at the cabin door and reduce the evacuation efficiency of the people in the cabin. Therefore, it is necessary to analyze the evacuation capacity of the escape slide, but the Pathfinder software does not support pedestrians escaping by The slide carries out the motion simulation of evacuation behavior by sliding down, and there are few evacuation experiments on aircraft escape slides, so the relevant slide flow experimental data has not been obtained yet. In the actual situation, there is a large section in the process of people sliding down the slide in the suspended state and doing parabolic motion, so the actual friction force is ~0. Based on this, when the passenger cabin is in a horizontal state, this paper takes the aircraft escape slide equivalent to a smooth slope with a depression angle of 40° to carry out the ideal calculation, and its equivalent schematic diagram is shown in Figure 11.

In this paper, it is assumed that the length of the slide is fixed at 7 m, and the pedestrian is accelerated by gravity from the top of the slide with an initial velocity of 0 at the exit of the passenger cabin at a height of 4.5 m from the ground, and its sliding acceleration *a* = *g* × sin[(40 × π)/180], from this, it is calculated that the time for the passenger to fall from the top of the slide to the bottom is about 1.49 s. Assuming that the next passenger immediately falls from the top after the previous passenger leaves the slide, ignoring the influence of other factors, it is calculated that The corresponding flow is 1 divided by 1.49 equals 0.68 ped/s. When the cabin is tilted forward, the calculation method of the evacuation flow of slides with different angles from the ground is the same.

In this paper, it is assumed that the length of the slide is fixed at 7 m, and the pedestrian is accelerated by gravity at the exit of the passenger cabin at the height of 4.5 m from the ground at the top of the slide with an initial velocity of 0. From this, it is calculated that the time for a passenger to fall from the top to the bottom of the slide is about 1.49 s. Assuming that the next passenger immediately falls from the top after the previous passenger leaves the slide, ignoring the influence of other factors, the corresponding flow rate is calculated to be 0.68 ped/s. Therefore, to consider the influence of the evacuation capacity of the slide on the evacuation efficiency of the aircraft, it is necessary to perform an equivalent calculation on the width of the hatch according to the calculated flow of the slide. In this section, a single-exit room scenario is established to analyze the evacuation flow of pedestrians under different Exit width conditions. The room size is 10 × 10 m, the personnel density is uniformly set to 2 ped/m^2^, and other personnel parameter settings are consistent with the above.

**Figure 12:**
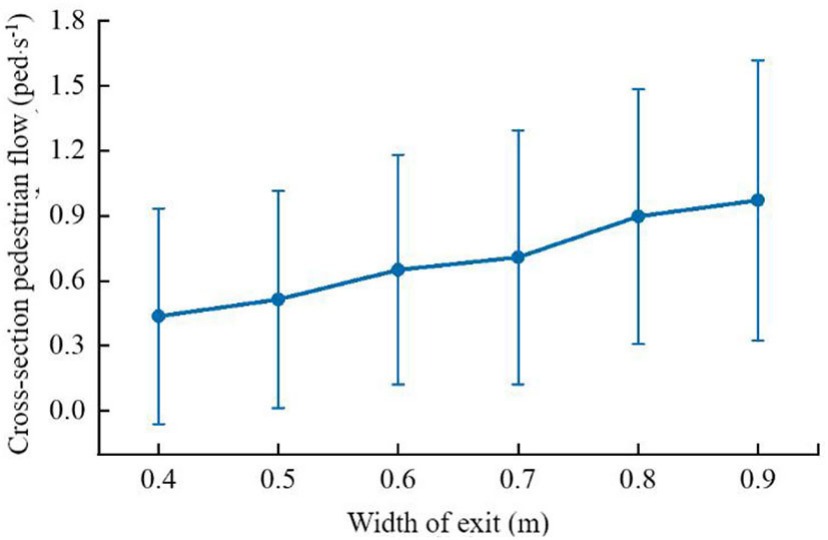
Outlet section flow corresponding to different outlet widths (mean ± standard deviation).

By setting different outlet widths (the width is shortened from 0.9 to 0.4 m in turn), the changes in the point section flow under the condition of different outlet widths are obtained, and the average evacuation section flow of different outlet widths is obtained according to the statistics of the outlet flow distribution, as shown in Figure 12.

It can be seen from the figure that when the width of the exit is 0.7 m, the average flow rate of the evacuation is close to 0.68 ped/s. Therefore, in this paper, considering the evacuation flow of passengers via the evacuation slide, the width of the exit of the passenger door is revised to 0.7. The equivalent width of m is used to carry out the evacuation simulation of the passenger aircraft when it is horizontal.

In the same way, after the airliner tilts forward, the distance between Exit 1 and Exit 2 from the ground is reduced to about 3 m. After calculation, the angle between the slide and the ground is 26°, and the time it takes for passengers to slide from the top to the bottom of the slide is About 1.80 s, the corresponding flow is calculated to be about 0.56 ped/s. The distance from Exit 7 and Exit 8 to the ground increases, about 5.8 m. After calculation, the angle between the slide and the ground is 56°, and the time for passengers to slide from the top of the slide to the bottom is about 1.31 s. Calculate the corresponding flow rate of About 0.80 ped/s. Therefore, this paper also considers the evacuation flow of passengers through the evacuation slide when the passenger aircraft is tilted forward, and the width of the passenger door Exit 1 and Exit 2 is corrected to an equivalent width of 0.5 m, and the width of the passenger door Exit 7 and Exit 8 is corrected to an equivalent width of 0.8 m, and the height of Exit 5 and Exit 6 does not change significantly when the passenger aircraft tilts forward, so the equivalent width of 0.7 m is still selected, and the following is the case after the passenger aircraft tilts forward? Personnel evacuation simulation.

**Figure 13:**
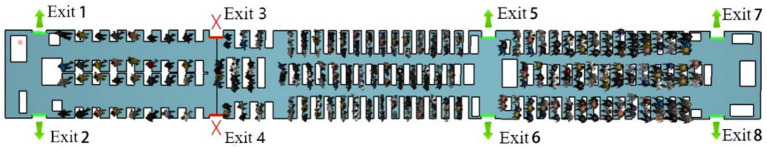
Schematic diagram of passenger cabin evacuation model.

By studying the internal structure of Airbus A350-900 wide-body passenger aircraft, evacuation dual aisles, door position and passenger seat distribution information, and related parameters, combined with other parameter settings above, the construction of the cabin personnel evacuation model is completed. The schematic diagram of the model is shown in Figure 13.

**Figure 14:**
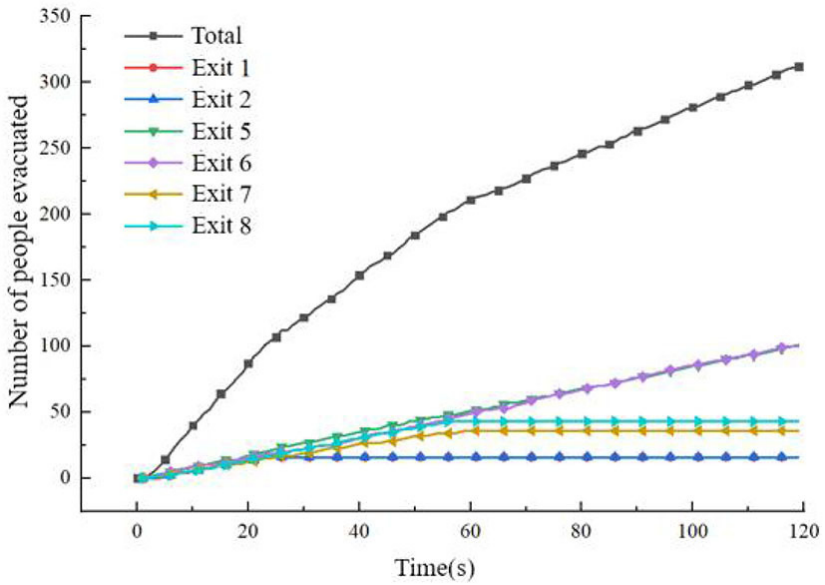
Statistics of the number of evacuees when the cabin is horizontal.

When the passenger cabin is in a horizontal state, the evacuation speed of personnel decreases to a certain extent in the middle of the evacuation, and the evacuation efficiency of passengers in the cabin is not high during some periods. The specific evacuation situation is shown in Figure 14.

**Figure 15:**
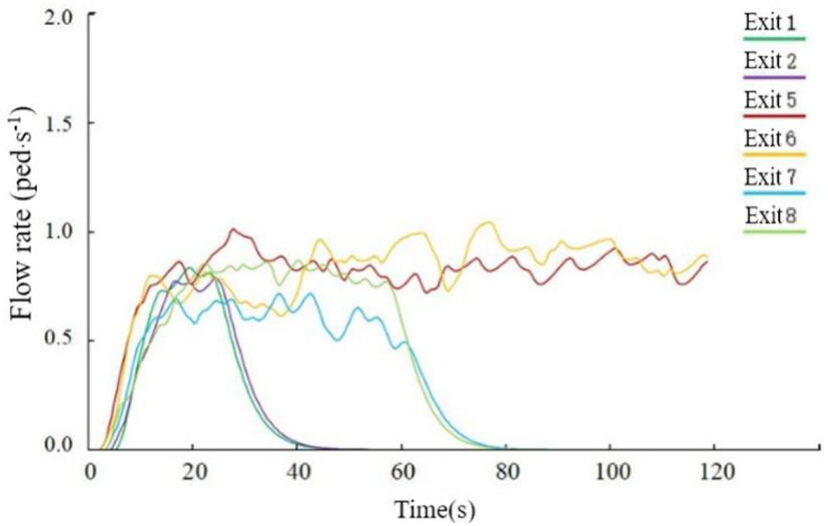
The flow of each outlet when the cabin is in a horizontal state.

As seen from Figure 14, in the middle of the evacuation, all passengers moved to the evacuation channel or near the evacuation exit, which caused a certain congestion phenomenon, resulting in a decrease in the evacuation rate. At about 25 s, the number of people evacuated at Exit 1 and Exit 2 no longer increased, and at about 55 s, The number of evacuees at Exit 7 and Exit 8 no longer increased, indicating that the above four exits were idle at the corresponding time, and the average utilization rate of the exits was not high. In the later evacuation period, the remaining passengers were evacuated only through Exits 5 and 6. The flow at the exit is shown in Figure 15. After 119.8 s, all 312 passengers were evacuated.

**Figure 16:**
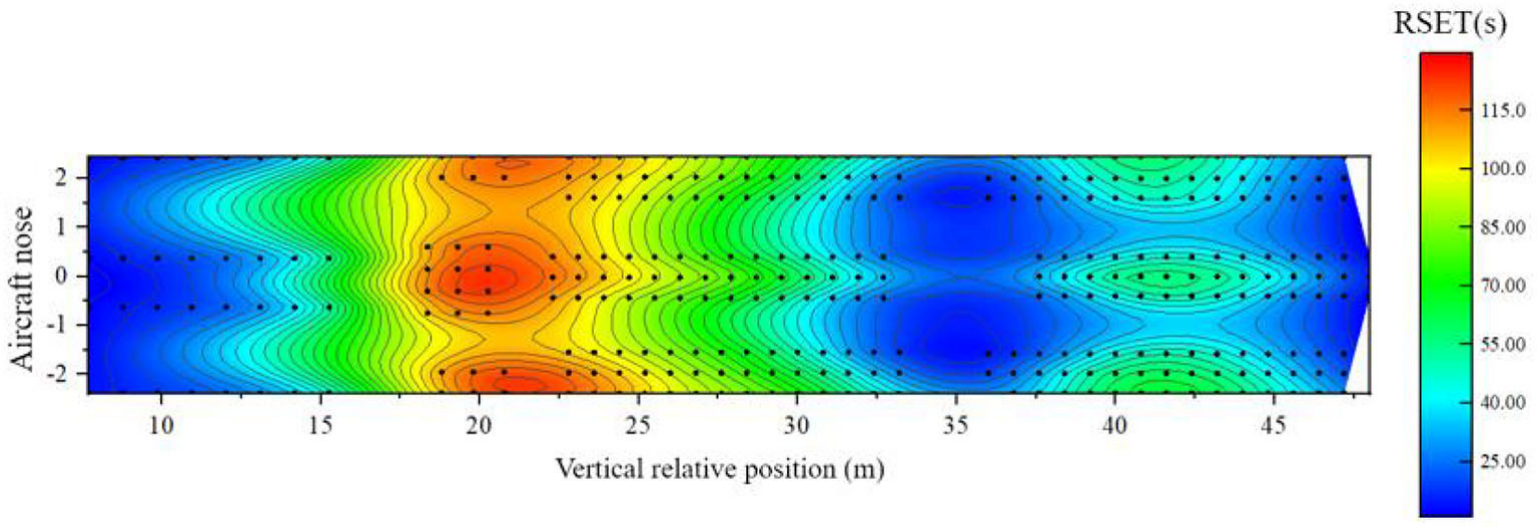
Distribution of necessary evacuation time when the cabin is horizontal.

The necessary evacuation time consists of three parts: early warning time, passenger response time, and movement time (24–26). Figure 16 shows the distribution of the necessary evacuation time for passengers at different seats in the passenger cabin when the passenger cabin is in a horizontal state under the condition of full load. The higher the evacuation efficiency of the individual, the shorter the time required for evacuation and the smaller the safety risk borne by the movement of the safety exit.

Combined with the analysis of the evacuation situation in the horizontal state of the aircraft, it can be seen from Figure 16 that:

(1) The RSET of passengers in business class is the smallest. All passengers use Exit 1 and Exit 2 for evacuation. The RSET of passengers in the cabin is basically within 25 s, which is the safest class in the passenger cabin, and the evacuation risk is relatively low;
(2) Passengers in the super economy class have the largest RSET. Under the most unfavorable evacuation conditions set in this paper, since exits 3 and 4 cannot be used for evacuation and escape, the super economy class is the farthest from the evacuation exit, and the people on the evacuation path are the farthest. The dense and crowded phenomenon is serious, and the RSET of a small number of passengers has even reached several times that of passengers in business class, which is the most dangerous class in the cabin, and the risk of evacuation is the highest;
(3) The main reason for the short RSET of business class is that business class with a passenger capacity of 32 people in the class with the most sparse distribution of people, and there are many evacuations exits available per capita. At the same time, the RSET of passengers in seats close to the exits also be significantly smaller than in other locations;
(4) Passengers in the middle seats of the same class in the cabin, especially those not adjacent to the aisle, have unfavorable factors for evacuation, such as long evacuation paths, dense distribution of people in the area, and small movement space, which lead to the evacuation of these passengers. It is easier to be congested, the evacuation time is longer, the evacuation efficiency is lower, and the evacuation risk is relatively higher.

**Figure 17:**
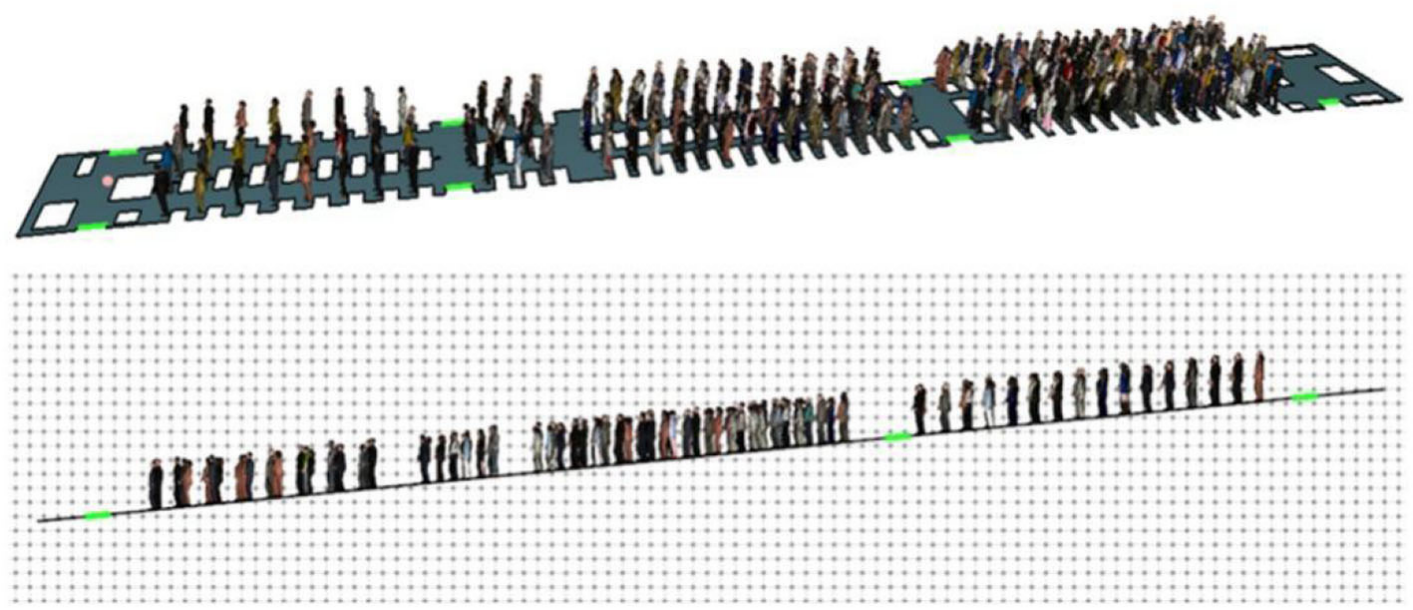
Schematic diagram of the model when the cabin is tilted forward.

Corresponding to the PyroSim fire model of the passenger aircraft cabin in the forward tilt state, the cabin is tilted forward 5.6°, the nose side touches the ground, and the schematic diagram of the cabin model in the forward tilt state is shown in Figure 17.

**Figure 18:**
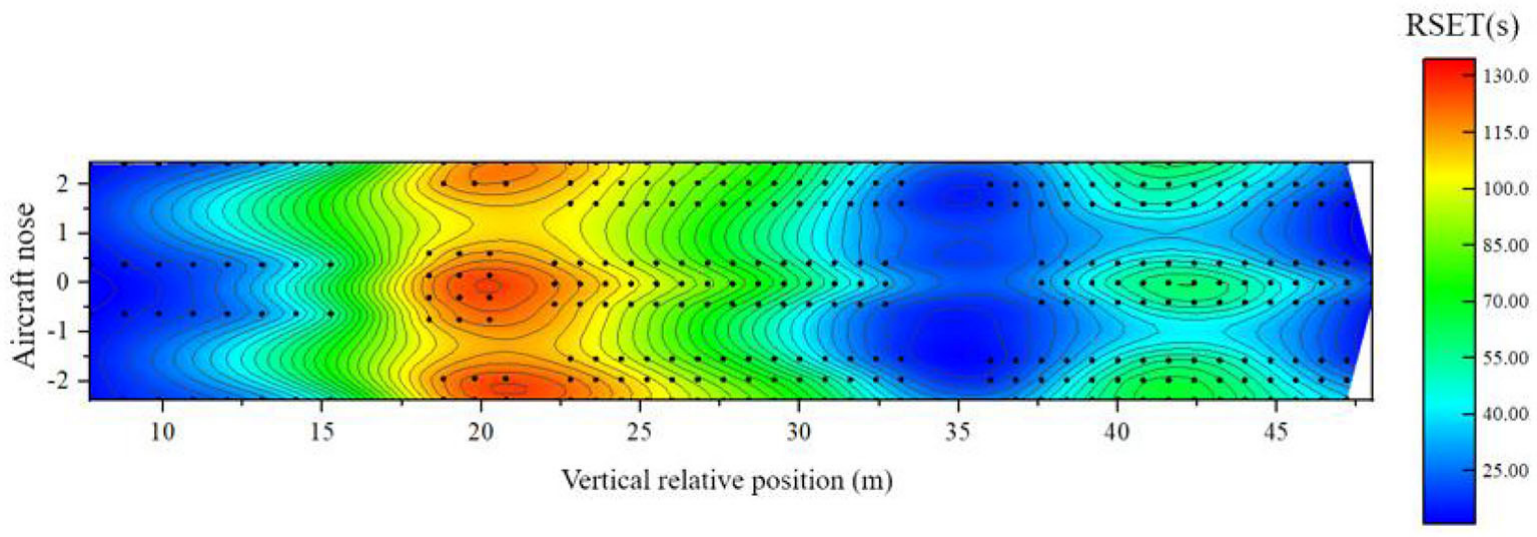
Distribution of necessary evacuation time when the cabin is tilted forward.

Figure 18 shows the distribution of necessary evacuation time for passengers at different seats in the cabin under full load when the passenger cabin is tilted forward.

Combined with the analysis of the evacuation situation when the passenger aircraft is tilted forward, it can be seen from Figure 18 that:

(1) The forward tilt angle of the cabin is 5.6°, which has little effect on the spatial distribution of the overall available evacuation time. Passengers in business class have the smallest RSET, which is basically within 25 s. Similar to when the cabin is in a horizontal state, business class is still a relatively safer class in the passenger cabin, with the lowest evacuation risk;
(2) Super economy class and close to fire sources Some passengers in economy class seats have the longest RSET, most of which are several times more than the RSET of passengers in business class. The necessary evacuation time for a small number of passengers is about 130 s. This part of the area is the most dangerous area in the passenger cabin. Evacuation risk is the highest.
(3) There is little difference between other evacuation conditions and when the cabin is in a horizontal state.

## Risk analysis of personnel safety evacuation

When ASET > RSET, that is, ASET-RSET > 0, personnel can evacuate safely before suffering adverse effects from emergencies. Define T_rem_ (T_rem_ = ASET – RSET) as the remaining evacuation time. When T_rem_ > 0, passengers can evacuate the area before being affected by the fire accident. The smaller T_rem_ is, the longer the passenger is exposed to the fire risk, and the greater the risk; the greater the T_rem_, the earlier the passenger is away from the danger brought by the fire, and the smaller the risk (27, 28).

### Distribution of remaining evacuation time

**Figure 19:**
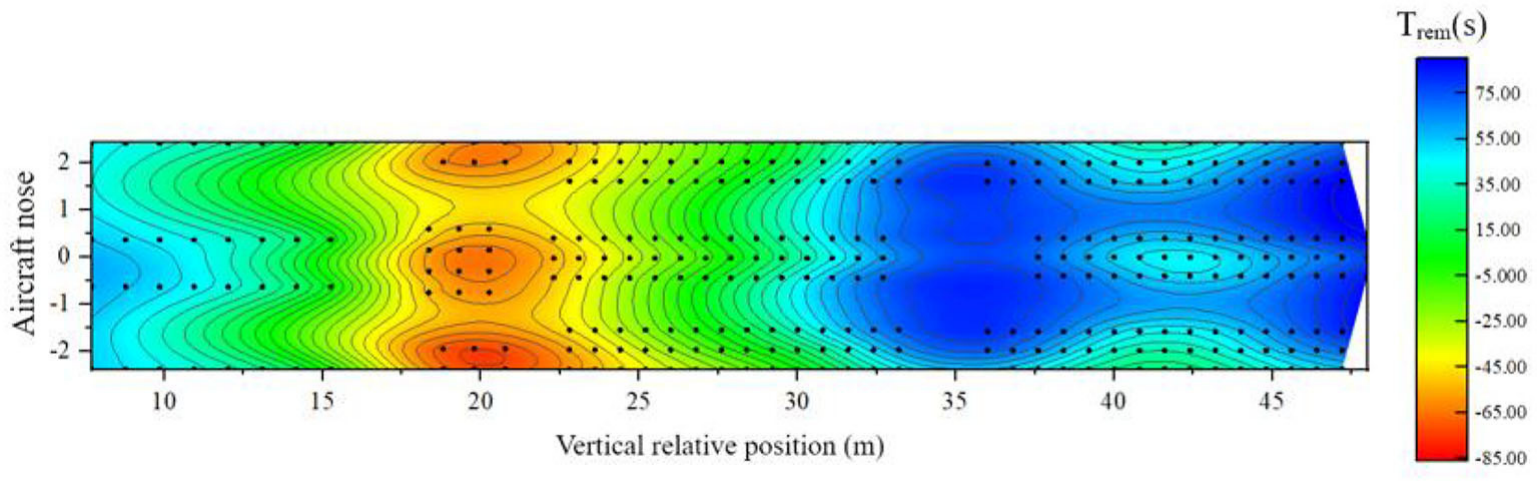
Distribution of remaining evacuation time when the cabin is horizontal.

When the airliner is in a horizontal state, there are 86 seats in the three classes of cabins, and the risk of safe evacuation is high. Figure 19 shows the distribution of the remaining evacuation time of passengers at different positions in the fully-loaded cabin.

Combined with the previous analysis of the cabin in a horizontal state, it can be seen from Figure 19 that:

(1) In the area of 6–16 m relative to the longitudinal position of the cabin: this area is the area where the business class seats are located, and the remaining evacuation time is about 15–55 s. between. Although the fire source is close to this area, the overall safe evacuation time is short, but the distribution of people is relatively sparse and there are many evacuation exits available per person, and the evacuation of people is faster and the RSET is shorter, so the overall safety is relatively low. There are 6 passengers in this area with a high risk of safe evacuation;
(2) In the 16–30 m area relative to the longitudinal position of the cabin: this area is the area where the super economy class and the economy class seats set in the middle of the cabin is located. The remaining evacuation time is <0 s, and the remaining evacuation time at some seats is even lower than – 65 s. It is the most unsafe area in the entire cabin. The main reason is that the area is close to the fire source, the ASET of most seats is short, and the failure of exit 4 resulted in a significant increase in the RSET of passengers in this area. There are 73 passengers in this area with a high risk of safe evacuation;
(3) In the area 30 m behind the relative longitudinal position of the cabin: this area is the area where the remaining economy class seats in the cabin are located, and some seats in this area are not adjacent to the aisle. The remaining evacuation time of the passengers is about – 5 s. Although this area is far from the fire source, the density of people is higher than that of other classes, resulting in a large load at the evacuation exit and clear congestion, resulting in a longer RSET for passengers in the middle. This layout The lower passenger ASET near the bulkhead is relatively short and more vulnerable to fire. Seven passengers in the area are at high risk for safe evacuation.

**Figure 20:**
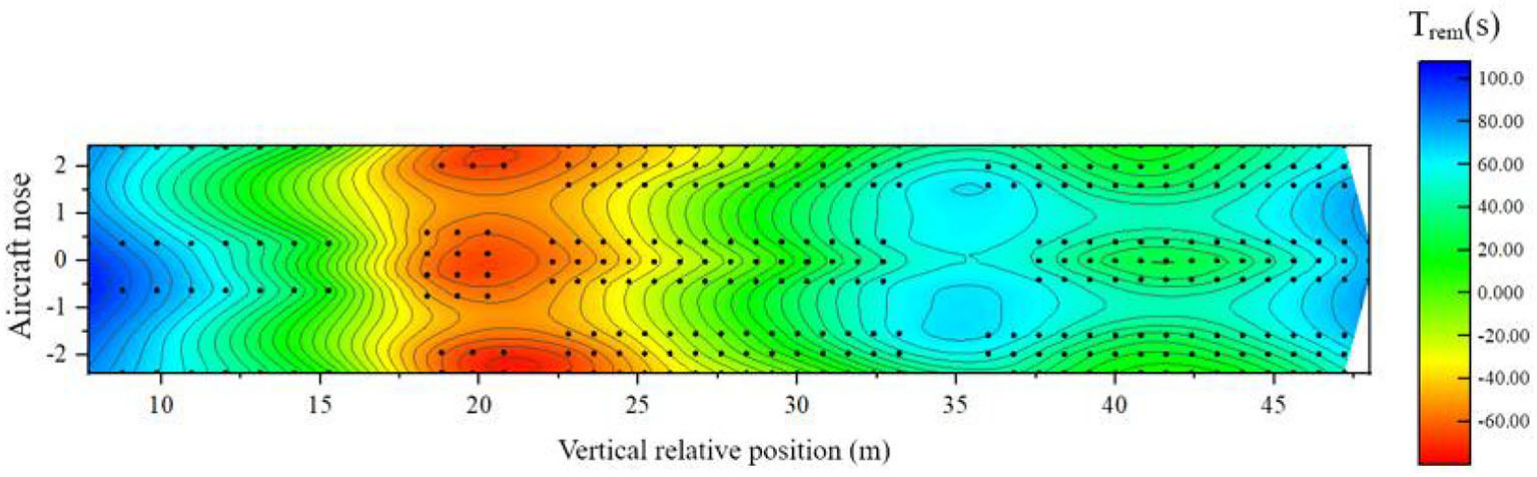
Distribution of remaining evacuation time when the cabin is tilted forward.

When the airliner is tilted forward, there are 97 seats in the three classes of cabins, with a high risk of safe evacuation. Figure 20 shows the distribution of the remaining evacuation time for passengers at different positions in the fully-loaded cabin.

(1) In the area of 6–13 m relative to the longitudinal position of the cabin: this area is the area where most of the business class seats are located near the nose side, and the remaining evacuation time of passengers is more than 40 s. Although the fire source is close to the area, the overall available safe evacuation time is short, but the distribution of people in this area is relatively sparse, and there are many evacuation exits available per person, which makes the evacuation of people fast. Spread, the seat ASET on the nose side away from the fire source is extended compared to the horizontal state. There are 4 passengers in this area with a high risk of safe evacuation;
(2) Within the 13–35 m area relative to the longitudinal position of the cabin: this area includes business class seats, super economy class seats, and economy class seats set in the middle of the cabin, which is close to the fire source. The remaining evacuation time in the area near the fire source is less than -the 40 s, and some seats are even less than -the 60 s. Passengers close to the bulkhead are affected by the fire more quickly. This area is the most unsafe in the entire cabin. There are 83 passengers in this area with a high risk of safe evacuation;
(3) In the area 35 m behind the relative longitudinal position of the cabin: this area is the area where the economy class seats are located in the rear section of the cabin, and the remaining evacuation time for passengers in the middle section of this section is About - 10 s, although the area is far away from the fire source, the speed of the smoke reaching the area is accelerated under the action of the forward tilt of the passenger aircraft, which leads to the shortening of ASET. Similarly, under the “3-3-3” horizontal layout of the seats. The area is a den, densely populated, the evacuation exit is heavily loaded, and the congestion is also obvious, resulting in a longer RSET for passengers far from the exit in the middle of the area. There are 10 passengers in the area with a high risk of safe evacuation.

To sum up, when the cabin is tilted forward, the safe evacuation risk in the cabin is higher than the risk when the cabin is in a horizontal state, and the evacuation situation when the cabin is tilted forward, and a fire occurs is a more dangerous situation than the conventional horizontal state. Therefore, putting forward a targeted cabin evacuation optimization scheme has important reference significance in the engineering practice in the field of aircraft safety.

### Optimal design scheme for evacuation

The evacuation pressure of Exit 5 and Exit 6 in the middle is high, and the utilization rate of the four cabin doors available for economy class passengers is uneven, resulting in different degrees of congestion, resulting in low overall evacuation efficiency. Given the above situation, it is proposed to add zoning guidance to rationally allocate the use of available evacuation exits instead of using the overall passenger to select exits based on the nearest principle. In two states, horizontal and forward, this section analyzes the optimization method in the horizontal state of the cabin.

**Figure 21:**
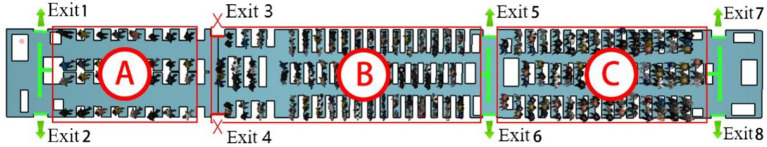
Schematic diagram of the evacuation optimization scheme for adding guidance.

The specific method is to divide the business class into area A, and guide all passengers in this area to evacuate to Exit 1 and Exit 2 during evacuation; divide the super economy class and the economy class set in the middle of the cabin into area B, and guide all passengers in this area during evacuation. Evacuate to Exit 5 and Exit 6; divide the remaining economy class into area C, and guide all passengers in this area to evacuate to Exit 7 and Exit 8 during evacuation. The specific guidance optimization scheme is shown in Figure 21.

**Figure 22:**
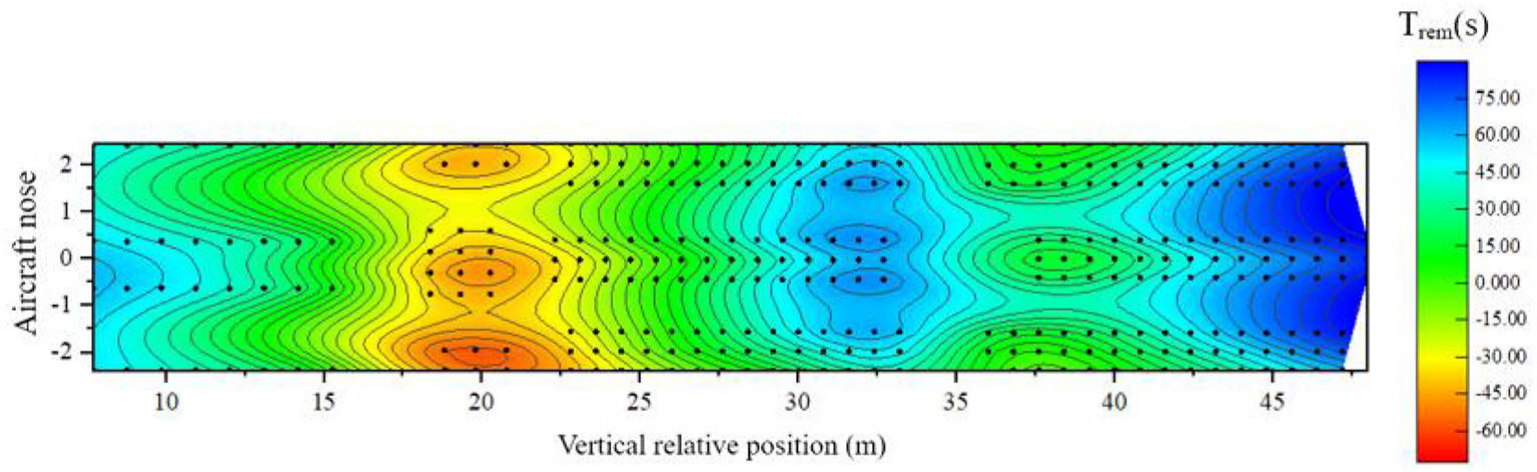
Distribution of remaining evacuation time after adding guidance.

**Figure 23:**
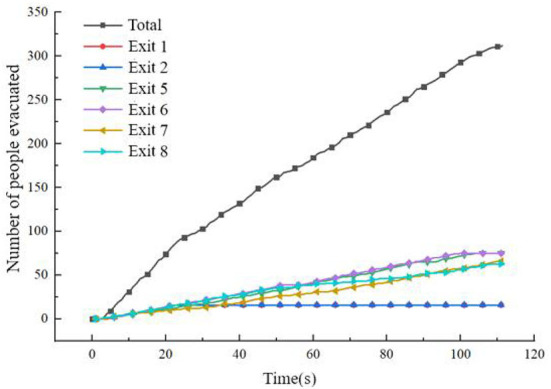
Statistics of evacuated people after adding guidance.

**Figure 24:**
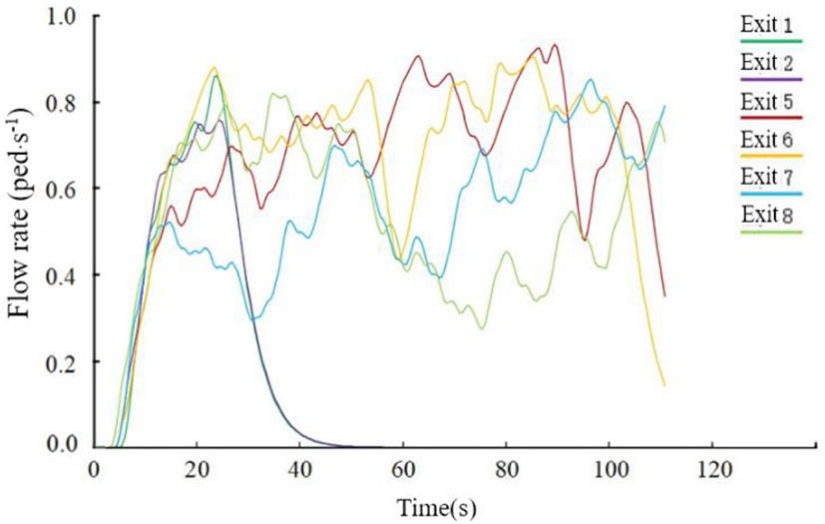
Each outlet flow after optimization.

The distribution of the remaining evacuation time after optimization is as follows:

It can be seen from Figure 22 that this scheme improves the evacuation efficiency of this part of the passengers by alleviating the congestion in the middle section of the cabin where the risk of cabin evacuation is the highest, effectively reducing the evacuation risk of passengers in area B, and optimizing the remaining cabin. Evacuation time distribution. The optimized cabin evacuation situation is shown in Figures 23, 24:

After adding evacuation guidance, the overall evacuation time of the cabin is reduced to 111.3 s compared with 119.8 s before optimization, and the reduction is 8.5 s. As seen from the above figure, the evacuation flow of Exit 5, Exit 6, Exit 7, and Exit 8 is more balanced, and the utilization rate of available exports improved. Passengers in area C no longer use Exit 5 and Exit 6 for evacuation, which reduces the evacuation pressure on the pair of exits and reduces the congestion of people near the pair of exits. After optimization, the evacuation situation in area A has not changed from the previous one. There are 6 passengers with a high risk of safe evacuation; 64 passengers in area B have a high risk of safe evacuation; 8 passengers in area C have a high risk of safe evacuation, with a total of 63 passengers. The risk of safe evacuation is high, a total of 8 people have been reduced compared with before optimization, and the reduction ratio is 9.3%.

## Conclusion

In this paper, by setting the fire scene in the cabin of the wide-body dual-aisle civil airliner Airbus A350-900, FDS software is used to simulate the fire development process caused by the second pair of mid-section aviation fuel leakage, and the impact of changes in relevant parameters on the cabin evacuation risk is analyzed. According to the time when the 312 observation points corresponding to each seat reach the critical value of any safety evaluation index, the available safe evacuation time of all seats is calculated, and the ASET distribution of the entire cockpit is obtained. Then, the Pathfinder software is used to simulate the evacuation process of passengers in both horizontal and forward states. When the passenger plane is fully loaded, the necessary safe evacuation time for passengers with 312 seats is calculated, and the RSET distribution of the entire cabin is obtained. According to the distribution of ASET and RSET, the distribution of the remaining evacuation time in the cabin is calculated, the safety risks of passengers in different positions in the cabin are analyzed, and an evacuation optimization design scheme for optimizing the distribution of the remaining evacuation time is proposed.

According to the above model simulation results and analysis, it can be seen that the safety hazards of the Airbus A350-900 wide-body passenger aircraft mainly include: when a serious fire occurs in the cabin, passengers may not be able to complete safety before a certain safety level index reaches a critical value. The main reasons for this phenomenon are: in the worst case, the passengers in the cabin far away from the available evacuation exits have long evacuation paths, long evacuation time, and long RSETs for these passengers. Passengers in the cabin close to the bulkhead are more vulnerable to fire in a fire situation, resulting in their short ASET.

The study found that the forward tilt of the cabin caused the effects of the fire to spread more quickly into the rear space of the cabin, and the tilt of the cabin also affected the evacuation of passengers. The cabin's forward tilt can significantly adversely affect safe evacuation.

## Data availability statement

The original contributions presented in the study are included in the article/supplementary material, further inquiries can be directed to the corresponding author/s.

## Author contributions

WL and CZ: funding acquisition. WL, CZ, and YY: validation. WL: conceptualization and writing—original draft. LX and JL: formal analysis, methodology, and revising. CZ: supervision. CZ and YY: revision discussion. CZ, YY, LX, and JL: writing—review and editing. All authors contributed to the article and approved the submitted version.

## Funding

This work was supported by the National Natural Science Foundation of China (Grant Nos. 52072286 and 72074149), the Opening Fund of the State Key Laboratory of Fire Science (Grant No. HZ2021-KF11), and the Fundamental Research Funds for the Central Universities (Grant Nos. 2022IVA108 and 2020VI002).

## Conflict of interest

Author YY was employed by Saifeite Engineering Technology Group Co., Ltd. The remaining authors declare that the research was conducted in the absence of any commercial or financial relationships that could be construed as a potential conflict of interest.

## Publisher's note

All claims expressed in this article are solely those of the authors and do not necessarily represent those of their affiliated organizations, or those of the publisher, the editors and the reviewers. Any product that may be evaluated in this article, or claim that may be made by its manufacturer, is not guaranteed or endorsed by the publisher.
